# Exploring the transcription factor activity in high-throughput gene expression data using RLQ analysis

**DOI:** 10.1186/1471-2105-14-178

**Published:** 2013-06-06

**Authors:** Florent Baty, Jochen Rüdiger, Nicola Miglino, Lukas Kern, Peter Borger, Martin Brutsche

**Affiliations:** 1 Division of Pulmonary Medicine, Cantonal Hospital St. Gallen, Rorschacherstrasse 95, CH-9007 St. Gallen, Switzerland; 2 Department of Biomedicine, University Hospital Basel, Petersgraben 4, CH-4001 Basel, Switzerland; 3 Pulmonary Medicine, Cantonal Hospital Zug, Landhausstrasse 11, CH-6340 Baar, Switzerland

## Abstract

**Background:**

Interpretation of gene expression microarray data in the light of external information on both columns and rows (experimental variables and gene annotations) facilitates the extraction of pertinent information hidden in these complex data. Biologists classically interpret genes of interest after retrieving functional information from a subset of genes of interest. Transcription factors play an important role in orchestrating the regulation of gene expression. Their activity can be deduced by examining the presence of putative transcription factors binding sites in the gene promoter regions.

**Results:**

In this paper we present the multivariate statistical method RLQ which aims to analyze microarray data where additional information is available on both genes and samples. As an illustrative example, we applied RLQ methodology to analyze transcription factor activity associated with the time-course effect of steroids on the growth of primary human lung fibroblasts. RLQ could successfully predict transcription factor activity, and could integrate various other sources of external information in the main frame of the analysis. The approach was validated by means of alternative statistical methods and biological validation.

**Conclusions:**

RLQ provides an efficient way of extracting and visualizing structures present in a gene expression dataset by directly modeling the link between experimental variables and gene annotations.

## Background

Gene expression microarray technology enables simultaneous monitoring of the expression level of thousands of genes. The biological interpretation of gene expression microarray findings remains challenging since it generally requires the explicit link to supplementary knowledge related to the function of genes and their inter-connections through functional networks [1].

Information on samples, usually related to the design of experiments (*e.g.* disease classes, treatment, time-course effect, replicates, *etc.*), is commonly integrated and modeled in the main analysis in order to identify genes which are specifically dysregulated under certain pre-defined conditions. In a second step, a selection of genes of interest is classically interpreted in the light of external information including functional annotations derived from various knowledge databases such as Gene Ontology [2] or KEGG (Kyoto Encyclopedia of Genes and Genomes) molecular pathways [3]. These two steps (gene extraction followed by interpretation) are generally distinct and come sequentially. We hypothesize that treating these two analytical steps in one single integrated manner can facilitate the interpretation of gene expression microarray data.

Transcription factors are regulatory elements which bind to specific DNA sequences generally located in the promoter region of genes. They orchestrate the regulation of genes by enhancing or inhibiting their transcription. Putative transcription factor binding sites (TFBS) can be predicted by searching for short specific motifs in the region upstream of the gene transcription starting site. Identifying TFBS in a list of genes of interest can help the interpretation of gene expression data in the light of the transcription factor activity. The prediction of TFBS in the promoter region of a list of genes was until recently a tedious task, involving the extraction of gene promoter sequences, followed by pattern recognition using motif databases such as TRANSFAC [4] or JASPAR [5]. However, recent bioinformatic developments allow the automation of most of these complex processes. Several open-source applications were developed, including statistical packages (*e.g.* the R package MotIV [6]), as well as various web tools. As an example, Zambelli and collaborators [7] recently proposed a new application — *pscan* — which facilitates the discovery of TFBSs, which are over- (or under-) represented in a list of genes.

Despite the emergence of novel bioinformatics solutions, methodological improvements are required in order to integrate TFBS information in the analytical work-flow of gene expression data and simplify results visualization and interpretation.

Correspondence analysis (CA) is, together with principal component analysis (PCA), a popular ordination method for the exploratory analysis of gene expression microarray data. Applications of CA in the field of ’omics was first described in the early 2000s [8-10]. Since then, several refinements of CA were described, exploiting some particular features of the method in order to investigate patterns of variation present in microarray data. Besides the table of direct interest (gene expression data), external information regarding both observations and genes is generally available. This information can be integrated to CA as shown by Busold and colleagues [11]. The authors proposed to use CA for the exploration of microarray data in the light of gene ontology annotations. This supplementary information is superimposed with the original CA results. CA eventually provides graphical solutions that allow to visualize in a single plot, genes, observations, experimental conditions and gene annotations [11,12]. On the other hand, a supervised counterpart of CA (a.k.a *constrained correspondence analysis*) was applied to the analysis of microarray data. Constrained CA has the advantage over unsupervised-CA of taking the external information explicitly into account. *Between-group correspondence analysis* (BGA) [13] is an example where an explanatory variable is used to constrain CA. BGA applies when observations are grouped into categories (*e.g.* disease classes) defined by one single nominal variable. BGA tries to best discriminate the per-group centroids by finding axes that maximize the ratio of between- over within-group variance. More complex designs of experiments can be modeled using the generalized *correspondence analysis with respect to instrumental variables* (CAIV) [14]. Qualitative as well as quantitative variables can be modeled, positively or negatively (effect removal), within the framework of CA. Recently, Jeffery and colleagues [15] combined BGA with an additional table including the occurrence of transcription factor binding sites (BG-COI) using co-inertia analysis [16,17].

In this manuscript, we introduce RLQ (R-mode; Q-mode; L-link between R and Q), to provide a broader generalization of the analysis of a central table of interest for which external information on both rows and columns is available. RLQ is a three-table ordination method, initially developed in ecological science [18,19]. Variations around the same *L*-structure principle exist in various fields of science such as food science (*L*-PLSR) [20], psychometry [21], consumer preference analysis [22]. In RLQ, the joint structure of three tables is analyzed, the central table being treated by CA.

RLQ analysis is suitable for answering questions such as: 

● How can we interpret gene expression data in the light of external gene annotations?

● How strong is the link between experimental variables and gene annotations?

● Can we find patterns of variation in the gene expression data set, which can be both explained by the sample and gene descriptors?

Throughout this work, we describe the general framework of RLQ and show its applicability for the interpretation of gene expression data in light of external gene annotations, with a main focus on the presence/absence of putative TFBS.

In the first section, the mathematical background of RLQ is described. As an illustration, RLQ is applied to a biological example where the aim is to explore the effect of the steroid mometasone furoate (MF) in the proliferation of primary human lung fibroblasts. The regulatory role of transcription factors in this example is of specific interest. It will be compared to the existing knowledge and challenged using alternative approaches. Additional biological validation will be provided. In the final section, the relevance of the method, as well as its strengths and limitations are discussed.

## Methods

### Theory of RLQ analysis

**Figure 1:**
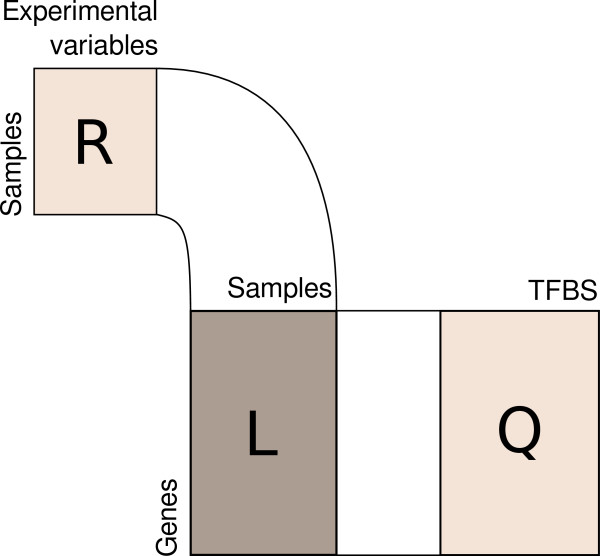
**Scheme of the RLQ analysis.** Three tables are involved: a central table (**L**) including the gene expression microarray data, and two tables (**R**) and (**Q**), including external information about rows (sample information, usually described by experimental variables) and columns (gene annotations, including *e.g.* the occurrence of transcription factor binding sites — TFBS), respectively.

RLQ was used to explore the inter-connections between three matrices linked together in an *L*-shape manner (Figure 1).

Let us define the following three tables: 

● **L** the (*n*×*m*) table of gene expression (*n* genes, *m* samples)

● **R** the (*m*×*p*) table including the experimental design information (*m* samples, *p* variables)

● **Q** the (*n*×*q*) table including external information about genes (*n* genes, *q* descriptors)

The inter-relationship between the three tables is analyzed by performing singular value decomposition (SVD) of the following: $R_{0}^{T}L_{0}^{T}Q_{0}$. The matrices (**R**_0_,**L**_0_,**Q**_0_) derive from the original (**R**, **L**, **Q**) matrices after the following transformations.

The central table **L** is treated by correspondence analysis (CA). In the scheme of CA, the table is converted into a *χ*^2^ distance matrix **L**_0_ defined as follows:

(1) $$L_{0}=D_{r}^{-1}(P-rc^{T})D_{c}^{-1}$$

with **P**=**L**/**N** the data matrix divided by its grand total, *r* the *n*-dim vector of row sums of **P**, *c* the *m*-dim vector of column sums of **P**, **D**_*r*_=*diag* (*r*) and **D**_*c*_=*diag* (*c*) the diagonal matrices of the row sums and the column sums respectively.

Let **Z** be the cross-product $Z=R_{0}^{T}L_{0}^{T}Q_{0}$. The singular value decomposition of **Z** can be written as follows:

(2) $$Z=U\Lambda V^{T}$$

with ***Λ*** the *k*×*k* (*k*= rank(**Z**)) diagonal matrix of singular values associated with **Z** with $\lambda_{1}\geq \cdots \geq \lambda_{k}>0$, **U** an (*m*×*k*) matrix whose columns are the left singular vectors of **Z** and **V** an *n*×*k* matrix whose columns are the right singular vectors of **Z**. The rows of **U** and **V** are orthogonal with respect to **D**_*r*_ and **D**_*c*_ respectively:

(3) $$U^{T}D_{r}U=V^{T}D_{c}V=I$$

The principal components and row coordinates are respectively given by $D_{r}^{-1/2}U$ and $D_{r}^{-1/2}U\Lambda$. The principal axes and column coordinates are respectively given by $D_{c}^{-1/2}V$ and $D_{c}^{-1/2}V\Lambda$.

Through this particular procedure, where the central table **L** is analyzed by correspondence analysis, RLQ analysis maximizes the covariance between linear combinations of columns of **R** and **Q**.

In order to test the link between the experimental design and the gene annotations, the fourth-corner statistic was used [19,23]. This permutation procedure tests the null-hypothesis of absence of link between tables **Q** and **R** mediated by**L**. The permutations are performed within each row of table **L**.

The transformation applied to **R** depends on the nature of the data (quantitative, qualitative, fuzzy coded, *etc.*). In the particular case where **R** only includes quantitative variables, **R**_0_ is obtained by normalizing **R** column-wise (centered by the weighted mean, and divided by the standard deviation). Row weights are set by the column weights of the previous CA procedure (*c*).

Similarly, the transformation applied to **Q** depends on the nature of the data. When **Q** only includes quantitative variables, **Q**_0_ is obtained by normalizing **Q** column-wise (centered by the weighted mean, and divided by the standard deviation). Row weights correspond to the row weights of the previous CA procedure (*r*).

### Transcription factor binding sites and functional annotations

TFBSs were extracted using the stand-alone version of the *pscan* software [7]. The original Affymetrix IDs needed first to be converted into RefSeq IDs. The following options were used: the TFBS database was TRANSFAC; the mapping was based on the promoter region specified as 450 bases upstream and 50 bases downstream the gene transcription starting site. *Pscan* outcome resulted in *z*-scores matrix linking each gene with each TFBS. High scores reflect a higher chance of presence of a given TFBS in the promoter region as compared to the genome-wide mean, whereas low scores reflect a lower chance of the presence of a given TFBS in a given gene promoter region as compared to the genome-wide mean. In turn, this *z*-scores matrix was used to build the occurrence matrix (table **Q**) in the RLQ analysis. The occurrence matrix reflects the presence/absence (0/1) of TFBS in a list of genes. We considered that *z*-scores superior to 2 reflects the actual presence of a given TFBS in the promoter region of the gene. The transformation of the quantitative *z*-score matrix into a qualitative occurrence matrix was decided in order to focus on TFBSs with the highest confidence. This resulted in a more sensitive analysis with a better readability of the results. The table of TFBS occurrence included 282 TFBS entries. Notice that some TFBS from the TRANSFAC database have several accession number corresponding to different motifs specific to the same TFBS. This explains why some TFBS are sometimes displayed more than once.

Molecular KEGG pathways and Gene Ontology annotations were directly retrieved using the hgu133a.db meta-data package. A table of KEGG term occurrence (table **Q**) was built based on the presence/absence of KEGG annotations for each of the investigated genes. Following this procedure, a total of 87 KEGG terms were specifically integrated. Similarly the table of GO terms occurrence (restricted to biological process domain) included 694 entries.

### Comparison with current standards

The results obtained by RLQ analysis were compared to TFBS enrichment analysis. Over-represented TFBS motifs were extracted using *pscan*. The TRANSFAC motif database was used with the same setting as the one used to generate the TFBS occurrence matrix in RLQ analysis. The TFBS enrichments are given by *z*-score test *p*-values. In addition, RLQ was compared with 3 competing ordination methods: CA, BG-COI and *L*-PLSR. The three ordination methods used in this comparison rely on three different schemes. Correspondence analysis is a 1-table ordination method, where supplementary information about rows and columns can be projected *a posteriori*. Between-group coinertia analysis is a 2-table ordination method where the main table (gene expression data) is constrained observation-wise by a single categorical variable defining groups among observations. *L*-PLSR is a 3-table ordination method where all three tables (gene expression data and its row/column external information) are treated symmetrically. All these methods were applied to explore the transcription factor activity associated with MF (including all 22’283 probe sets). Their relative performance was assessed and compared to RLQ.

### Mometasone furoate dataset

In this dataset, we investigated the time-course effect of the glucocorticoid mometasone furoate (MF) on primary human lung fibroblasts. Two cell lines of fibroblasts were established from pulmonary biopsies obtained from patients undergoing lobectomie or pneumonectomy for peripheral lung cancer as described elsewhere [24]. Cells were cultured in RPMI 1640, 5% FCS, 20 mM HEPES or DMEM, 10% FCS, MEM vitamins. All cell culture media and additives were purchased from Facola/Seromed (Basel, Switzerland). Treatment and experiments were performed between passages 2 and 5. Before the preparation of nuclear and cytosolic extracts, cells were subcultured in Petri dishes and kept for 24 to 48 h in serum-rich medium until they reached 60-80% confluency. Prior to treatment, cells were serum deprived for 24 h with 0.1% FCS. Low serum medium was exchanged every 12 h. MF was diluted in 100% ethanol and added to the medium with a final concentration of 10^−8^M. Cell lines were harvested and gene profiled at 8 time points (baseline, 20’, 40’, 1 h, 1.5 h, 2 h, 3 h and 6 h). The gene expression level was monitored according to the manufacturer recommendations using the Affymetrix Genechip Human Genome U133A platform which measured 22’283 probe set intensity levels (raw files have been deposited in NCBI’s Gene Expression Omnibus (GEO) and are accessible through GEO series accession number GSE30242).

### Statistical considerations and implementations

All calculations were done using the **R** statistical software including the package ade4[25], as well as packages from Bioconductor [26] including affy, hgu133a.db, annotate, seqinr, GO.db. Gene expression data were normalized using the robust multichip average (RMA) method [27].

The RLQ procedure is implemented in the package ade4 with the function *rlq*. It requires the use of three separate analyses of tables **R**, **L** and **Q** which are combined using the function *rlq*. As previously mentioned, the central table **L** must be treated by CA, whereas the analysis of the two other tables (**R** and **Q**) depends on the nature of the variables (principal component analysis for quantitative variables, multiple correspondence for qualitative variables, Hillsmith analysis for a mixed table of quantitative and qualitative variables, etc.).

The ade4 package also includes Monte-Carlo permutation tests specifically implemented for *rlq*, as well as the function *fourthcorner* which is the implementation of the fourth-corner statistic measuring and testing the link between the three tables [19,23].

A wrap-up package (R package rlqomics) that helps to automate these analytical steps in the frame of genomics analysis was developed and is available upon request.

## Results

### Time course effect of mometasone furoate on the proliferation of lung fibroblasts

To provide a concrete application of RLQ, we analyzed the time course effect of the glucocorticoid MF on the proliferation of primary human lung fibroblasts in the light of transcription factor activity. Two cell lines treated by MF were monitored at 8 time points.

The genes which were mostly dysregulated during the time-course of MF treatment are described in Table 1. These genes included among others various key genes associated with the general mechanisms of the known action of glucocorticoids, such as the inhibition of the transcription of proinflammatory genes via specific transcription factors [28]. Accordingly, our results showed that MF down-regulated genes involved in the initiation and maintenance of inflammation, *e.g.* chemokines (CCL2, CCL7, CCRL1, CXCL2, CXCL3), interleukines (IL6, IL8, IL11, IL33), early response genes (EGR1, IER2, IER3) and transcription factors such as PI3K or FOXO1.

**Table 1:** List of the 100 genes mostly associated with the time-course effect of mometasone furoate on the proliferation of fibroblasts

**AffyID**	**RefSeq**	**Symbol**	**Name**
204908_s_at	NM_005178	BCL3	B-cell CLL/lymphoma 3
207510_at	NM_000710	BDKRB1	bradykinin receptor B1
221530_s_at	NM_030762	BHLHE41	basic helix-loop-helix family, member e41
210538_s_at	NM_001165	BIRC3	baculoviral IAP repeat containing 3
209183_s_at	NM_007021	C10orf10	chromosome 10 open reading frame 10
209182_s_at	NM_007021	C10orf10	chromosome 10 open reading frame 10
218723_s_at	NM_014059	C13orf15	chromosome 13 open reading frame 15
216598_s_at	NM_002982	CCL2	chemokine (C-C motif) ligand 2
208075_s_at	NM_006273	CCL7	chemokine (C-C motif) ligand 7
220351_at	NM_016557	CCRL1	chemokine (C-C motif) receptor-like 1
219343_at	NM_017913	CDC37L1	cell division cycle 37 homolog (S. cerevisiae)-like 1
209112_at	NM_004064	CDKN1B	cyclin-dependent kinase inhibitor 1B (p27, Kip1)
213006_at	NM_005195	CEBPD	CCAAT/enhancer binding protein (C/EBP), delta
206100_at	NM_001005502	CPM	carboxypeptidase M
209774_x_at	NM_002089	CXCL2	chemokine (C-X-C motif) ligand 2
207850_at	NM_002090	CXCL3	chemokine (C-X-C motif) ligand 3
202887_s_at	NM_019058	DDIT4	DNA-damage-inducible transcript 4
208892_s_at	NM_001946	DUSP6	dual specificity phosphatase 6
208891_at	NM_001946	DUSP6	dual specificity phosphatase 6
208893_s_at	NM_001946	DUSP6	dual specificity phosphatase 6
218995_s_at	NM_001168319	EDN1	endothelin 1
201694_s_at	NM_001964	EGR1	early growth response 1
201693_s_at	NM_001964	EGR1	early growth response 1
204560_at	NM_001145775	FKBP5	FK506 binding protein 5
202724_s_at	NM_002015	FOXO1	forkhead box O1
202723_s_at	NM_002015	FOXO1	forkhead box O1
209990_s_at	NM_005458	GABBR2	gamma-aminobutyric acid (GABA) B receptor, 2
217077_s_at	NM_005458	GABBR2	gamma-aminobutyric acid (GABA) B receptor, 2
204457_s_at	NM_002048	GAS1	growth arrest-specific 1
210002_at	NM_005257	GATA6	GATA binding protein 6
221577_x_at	NM_004864	GDF15	growth differentiation factor 15
200648_s_at	NM_001033044	GLUL	glutamate-ammonia ligase
217202_s_at	NM_001033044	GLUL	glutamate-ammonia ligase
209170_s_at	NM_001001994	GPM6B	glycoprotein M6B
206432_at	NM_005328	HAS2	hyaluronan synthase 2
38037_at	NM_001945	HBEGF	heparin-binding EGF-like growth factor
203821_at	NM_001945	HBEGF	heparin-binding EGF-like growth factor
215933_s_at	NM_002729	HHEX	hematopoietically expressed homeobox
204689_at	NM_002729	HHEX	hematopoietically expressed homeobox
204512_at	NM_002114	HIVEP1	human immunodeficiency virus type I enhancer binding protein 1
208808_s_at	NM_001130688	HMGB2	high-mobility group box 2
213844_at	NM_019102	HOXA5	homeobox A5
202081_at	NM_004907	IER2	immediate early response 2
201631_s_at	NM_003897	IER3	immediate early response 3
206924_at	NM_000641	IL11	interleukin 11
209821_at	NM_001199640	IL33	interleukin 33
205207_at	NM_000600	IL6	interleukin 6 (interferon, beta 2)
211506_s_at	NM_000584	IL8	interleukin 8
202859_x_at	NM_000584	IL8	interleukin 8
203126_at	NM_014214	IMPA2	inositol(myo)-1(or 4)-monophosphatase 2
213817_at	NM_001142523	IRAK3	interleukin-1 receptor-associated kinase 3
209184_s_at	NM_003749	IRS2	insulin receptor substrate 2
209185_s_at	NM_003749	IRS2	insulin receptor substrate 2
201466_s_at	NM_002228	JUN	jun proto-oncogene
201464_x_at	NM_002228	JUN	jun proto-oncogene
201473_at	NM_002229	JUNB	jun B proto-oncogene
213005_s_at	NM_015158	KANK1	KN motif and ankyrin repeat domains 1
203543_s_at	NM_001206	KLF9	Kruppel-like factor 9
203542_s_at	NM_001206	KLF9	Kruppel-like factor 9
205266_at	NM_002309	LIF	leukemia inhibitory factor (cholinergic differentiation factor)
218816_at	NM_018214	LRRC1	leucine rich repeat containing 1
219573_at	NM_001173977	LRRC16A	leucine rich repeat containing 16A
204389_at	NM_000240	MAOA	monoamine oxidase A
204918_s_at	NM_004529	MLLT3	myeloid/lymphoid or mixed-lineage leukemia (trithorax homolog, Drosoph
217546_at	NM_176870	MT1M	metallothionein 1M
206814_at	NM_002506	NGF	nerve growth factor (beta polypeptide)
212240_s_at	NM_181504	PIK3R1	phosphoinositide-3-kinase, regulatory subunit 1 (alpha)
212249_at	NM_181504	PIK3R1	phosphoinositide-3-kinase, regulatory subunit 1 (alpha)
212239_at	NM_181504	PIK3R1	phosphoinositide-3-kinase, regulatory subunit 1 (alpha)
201939_at	NM_006622	PLK2	polo-like kinase 2
204286_s_at	NM_021127	PMAIP1	phorbol-12-myristate-13-acetate-induced protein 1
204285_s_at	NM_021127	PMAIP1	phorbol-12-myristate-13-acetate-induced protein 1
209815_at	NM_000264	PTCH1	patched 1
204748_at	NM_000963	PTGS2	prostaglandin-endoperoxide synthase 2 (prostaglandin G/H synthase and
204338_s_at	NM_001102445	RGS4	regulator of G-protein signaling 4
204337_at	NM_001102445	RGS4	regulator of G-protein signaling 4
204339_s_at	NM_001102445	RGS4	regulator of G-protein signaling 4
204802_at	NM_001128850	RRAD	Ras-related associated with diabetes
204900_x_at	NM_003864	SAP30	Sin3A-associated protein, 30kDa
204614_at	NM_001143818	SERPINB2	serpin peptidase inhibitor, clade B (ovalbumin), member 2
209681_at	NM_006996	SLC19A2	solute carrier family 19 (thiamine transporter), member 2
203908_at	NM_001098484	SLC4A4	solute carrier family 4, sodium bicarbonate cotransporter, member 4
209884_s_at	NM_003615	SLC4A7	solute carrier family 4, sodium bicarbonate cotransporter, member 7
210286_s_at	NM_003615	SLC4A7	solute carrier family 4, sodium bicarbonate cotransporter, member 7
203372_s_at	NM_003877	SOCS2	suppressor of cytokine signaling 2
202935_s_at	NM_000346	SOX9	SRY (sex determining region Y)-box 9
202936_s_at	NM_000346	SOX9	SRY (sex determining region Y)-box 9
204597_x_at	NM_003155	STC1	stanniocalcin 1
204731_at	NM_001195683	TGFBR3	transforming growth factor, beta receptor III
220486_x_at	NM_017698	TMEM164	transmembrane protein 164
206025_s_at	NM_007115	TNFAIP6	tumor necrosis factor, alpha-induced protein 6
206026_s_at	NM_007115	TNFAIP6	tumor necrosis factor, alpha-induced protein 6
204933_s_at	NM_002546	TNFRSF11B	tumor necrosis factor receptor superfamily, member 11b
204932_at	NM_002546	TNFRSF11B	tumor necrosis factor receptor superfamily, member 11b
202478_at	NM_021643	TRIB2	tribbles homolog 2 (Drosophila)
208763_s_at	NM_001015881	TSC22D3	TSC22 domain family, member 3
206796_at	NM_001204869	WISP1	WNT1 inducible signaling pathway protein 1
205883_at	NM_001018011	ZBTB16	zinc finger and BTB domain containing 16
207513_s_at	NM_003452	ZNF189	zinc finger protein 189
220987_s_at			

The following information is included: Affymetrix identifiers, RefSeq identifiers, Gene symbols and complete gene description. For convenience, rows were ordered alphabetically according to gene symbols.

### RLQ analysis and transcription factors activity

**Figure 2:**
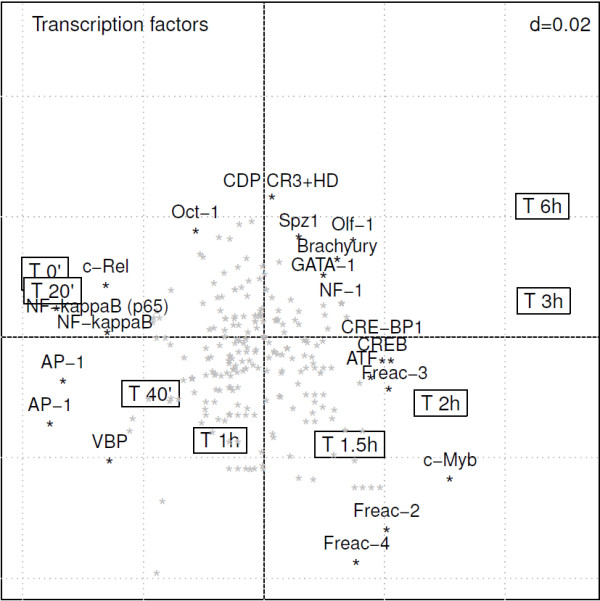
**RLQ analysis of the time course effect of mometasone furoate in the light of the activity of transcription factors.** This biplot representation (2 first axes of RLQ analysis) displays the TFBSs which are over-represented at specific time points. Framed labels show the time course. The transcription factors are displayed as stars, among which the 20 most influential are labeled. The upper right *d* value displays the grid scale.

The presence of putative TFBSs was assessed using *pscan*. In this example, RLQ analysis was based on the 100 genes mostly dysregulated during the time course effect of MF (Table 1). The transcription factors activity described below is measured by the presence of TFBSs that presumably belong (according to *pscan* predictions) to the promoter region of the most dysregulated genes. The putative transcription factors activity is summarized by an RLQ biplot (Figure 2). The biplot representation depicts the activity of transcription factors varying over time, as measured on the first 2 axes of RLQ. Biplots in RLQ should be interpreted similarly to biplots of other ordination methods. Both variables (TFBS) and observations (time points) are displayed. The TFBSs that mostly explain the variability extracted by the RLQ axes are the ones with the highest absolute scores (TFBSs located at the extremity of these axes). The further away the TFBSs are from the origin of the axes, the stronger they are associated with the time points lying towards the same direction. The cosine of the angle formed by two TFBSs indicates the correlation between these two TFBSs. Two TFBSs located in the opposite direction in the biplot (relatively to the origin of the axes) are inversely correlated.

The first 2 axes of the RLQ analysis summarize the vast majority of the total inertia (83% on the first axis and 14% on the second). RLQ analysis shows a gradient in the time course effect of MF associated with the first RLQ axis. Early time points correlate with low scores on the first axis whereas late time points correlate with high scores on the first axis. The second axis separates the intermediate to the extreme time points. When considering the 2 axes together, the different time points are distributed according to a U-shape. This effect, known as horseshoe effect, outlines the transcriptional changes over time as the effect of the action of MF. Although this effect results of a distortion of the ordination diagram, it facilitates the biplot-based interpretation of the activated TF along a unidirectional time gradient. Considering that the activity of MF is effective within 1-2 hours, the effect of MF can be subdivided into early and late effects. In Figure 2, the TFBSs associated with genes that are early up-regulated (time 1.5-2 h) are located in the lower right quadrant, whereas TFBSs associated with genes that are late up-regulated (time 3-6 h) are located in the upper right quadrant of the biplot. At the opposite directions, one can identify TFBSs which are associated with genes that are early down-regulated (upper left quadrant), as well as TFBSs associated with genes that are late down-regulated (lower left quadrant).

The forkhead-related activator 2, 3, and 4 (Freac 2, Freac 3, Freac 4), as well as cMyb are TFBSs which are prominently present in genes that are early up-regulated due to the effect of MF. The transcription factor c-Myb is known to play an important role in the regulation of cellular proliferation and differentiation. CREB, CRE-BP1, NF-1 (nuclear factor 1) are transcription factors which are associated with late up-regulated genes by MF. In the opposite direction (left quadrants), TFBSs which were present in down-regulated genes included AP1-2, transcription factors involved in cellular differentiation, proliferation and apoptosis. In Figure 2, NF- *κ*B and cRel were both found in the opposite direction (upper-left corner) from the early time points (times 1.5-2 h), suggesting an early inhibition of this transcription factor by MF. The combined role of NF- *κ*B and AP-1 transcription factors in the action of steroids is well documented in the literature [29,30]. As confirmed by the RLQ analysis, the repression of these transcription factors corresponds to the mechanism underlying the anti-inflammatory efficacy of corticosteroids [31,32]. Our data suggest that the octamer transcription factor 1 (OCT-1) is involved in the early down-regulation of genes by MF. Prior work indicated that OCT-1 cooperates synergistically with the glucocorticoid receptor (GR) in restricting transcriptional cooperativity to promoters containing DNA binding sites for both factors [33].

A permutation test based on the total inertia computed by RLQ analysis showed a significant link between **R** and **Q** through **L** (*p*<0.001). Another measure of the link between the three tables was further computed using the fourth-corner statistics. This procedure provides a synthetic representation of the TFBS activity over time (Additional file 1: Figure S1).

### Biological validation of the transcription factor activity

**Figure 3:**
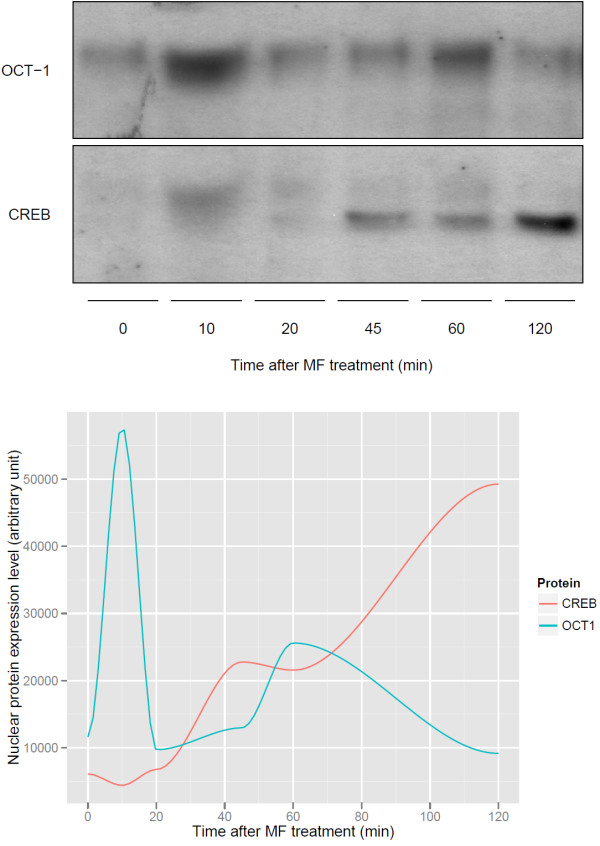
**Biological validation of the time course effect of mometasone furoate in the OCT-1 and CREB expression level.** The upper panel shows the expression levels of nuclear OCT-1 and CREB in primary human lung fibroblasts. The lower panel displays the kinetics of nuclear OCT-1 and CREB protein levels. Data shown are typical for three independently performed experiments.

The activity of transcription factors OCT-1 and CREB was further investigated using protein analysis by Western blotting. Based on our RLQ findings, OCT-1 and CREB have a strong activity associated with MF treatment. According to predictions summarized in the RLQ biplot (Figure 2), the high level of OCT-1 activity at baseline decreased over time, whereas CREB activity showed an increase within 2 hours. Figure 3 shows the protein expression time course (0-2 hours) of OCT-1 and CREB in the nuclear compartment of primary lung fibroblasts treated by MF. OCT-1 is present in the nucleus at early time points and is rapidly decreasing after steroid treatment. In addition, by comparing the protein expression level in the nucleus and the cytosol (Additional file 1: Figure S2), one can see that OCT-1 is decreasing from both compartments. This decrease of OCT-1 in the cytosol could be explained by either an increase of protein degradation or a reduction of *de novo* synthesis. In contrast, CREB is not present in the nucleus (only in the cytosol) at early time points, and after 45 min of MF treatment, it is translocated into the nucleus. Here MF acts predominantly by translocating the CREB protein, since the total of nuclear plus cytosolic CREB levels remains constant (indicating no significant netto cytosolic protein degradation) (Additional file 1: Figure S2). It is notable that the expression of OCT-1 increased after 60 minutes. However, protein analysis suggested that this occurs because of a decreasing level of cytosolic protein. Overall, MF induces a reduction of OCT-1 protein levels, and it induces a translocation of activated CREB within the first 2 hours, which is in agreement with RLQ predictions.

### RLQ analysis and other publicly available gene annotations

**Figure 4:**
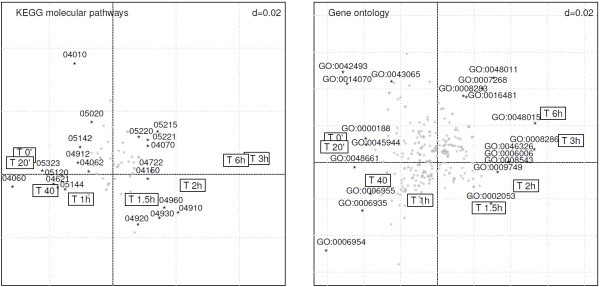
**RLQ analysis of the time-course effect of MF in the light of publicly available information (biplot representations on the 2 first RLQ axes).** The left panel provides an overview of the KEGG molecular pathways associated with the time course effect of mometasone furoate. The right panel displays the relevant GO terms that best relate to the time course effect of mometasone furoate. The framed labels display the time course, whereas KEGG and GO terms are represented as stars, among which the 20 most influential are labeled. The upper right *d* value displays the grid scale.

The same data set was analyzed in the light of other functional feature databases including KEGG biochemical pathways and Gene Ontology (GO). Figure 4 summarizes the RLQ analyses made at these 2 additional levels.The left panel displays the KEGG analysis of the MF dataset. The interpretation of the biplot follows the same process as for the TFBS analysis. Down-regulated genes (left quadrants) involve cytokine-cytokine receptor interactions pathways (KEGG 04060), whereas genes that are early up-regulated (lower right quadrant) are involved in pathways such as Type II diabetes mellitus (KEGG 04930) and insulin signaling pathway (KEGG 04910).

The right panel displays the GO analysis of the MF dataset. Genes down-regulated due to the effect of MF are associated with response to drug (GO:0042493) or response to organic cyclic compound (GO:0014070) (upper-left quadrant). After one hour, MF affects genes involved in inflammatory response (GO:0006954) and signal transduction (GO:0007165). At later time points (upper-right quadrant), genes that belong to functional categories such as insulin receptor signaling pathways (GO:0008286) and phosphotidylinositol-mediated signaling (GO:0048015) are over-represented.

### Comparison of RLQ analysis with alternative approaches

#### Enrichment analysis

The prediction of TFBS motifs in our MF dataset was analyzed using enrichment analysis (EA). The *pscan* software tool was used to identify over-represented TFBS motifs in the previously described list of 100 most dysregulated genes (Table 1). The 20 most significantly over-represented TFBS (*p*<0.1) were compared to the 20 most influential TFBS identified by RLQ and highlighted in Figure 2. Table 2 provides a summary of the TFBS motifs identified by either of the 2 methods. Common findings revealed by the 2 methods include the prominent role played by the NF- *κ*B/Rel transcription factor family. Among the transcription factors specifically extracted by RLQ, the forkhead family of transcription factors (FREAC2, FREAC3, FREAC4), regulating the expression of genes involved in cell proliferation, is activated in intermediate time points. Other TFBS specifically highlighted by RLQ are the transcription factors CREB, cMyb and CRE-P1 which are known to interact in the mechanisms of action of glucocorticoids. It is worth noting that the 2 transcription factors validated by Western blotting could not be identified using enrichment analysis.

**Table 2:** Enrichment analysis of transcription factor binding sites

**TFBS**	**Enrichment Analysis (*p*-value)**	**RLQ**	**Description**
AP-1 (C)		×	Activator protein 1. Signal transduction cascade mediated by glucocorticoids [30]
AP-1 (Q6)		×	—
AP-2 (Alpha)	× (0.0853)		Activating-protein 2. Regulatory roles in early development, apoptosis and cell-cycle control
AP-2 (Q6)	× (0.0618)		—
ATF		×	Activating transcription factor (class of AP-1 transcription factors dimers).
Brachyury		×	Transcription factor over-expressed in numerous lung tumor. Known to mediate epithelial-mesenchymal transition and promote invasion.
CDPCR3HD		×	Cut-like homeodomain protein
c-Myb		×	Myb proto-oncogene protein. Regulation of cell proliferation/differentiation, regulation of human glucocorticoid receptor [34]
CRE-BP1		×	Activating transcription factor 2. <Activation of transcription by interaction with glucocorticoid response elements [33]
CREB		×	cAMP response element-binding protein.
c-Rel	× (0.0009)	×	Proto-oncogene c-Rel. Member of the NF- *κ*B family.
Freac2		×	Forkhead-related activator. Differential activation of lung specific genes. Involved in cell growth/proliferation mechanisms
Freac3		×	—
Freac4		×	—
GATA-1		×	GATA-binding protein 1 (globin transcription factor 1). Involved in cell growth, cancer.
MAZR	× (0.0006)		Zinc finger protein related factor.
MZF1 (01)	× (0.0012)		Myeloid zinc finger protein. Control of cell proliferation.
MZF1 (02)	× (0.0196)		—
NF1		×	Nuclear factor 1. Chromatin remodeling and transcriptional activation.
NF- *κ*B	× (0.0043)		Nuclear factor *κ*B. Anti-inflammatory action of steroids [31].
NF- *κ*B 50	× (0.0035)		—
NF- *κ*B 65	× (<.0001)	×	—
NF- *κ*B C	× (0.0317)		—
NF- *κ*B Q6	× (0.0027)	×	—
Oct-1		×	Octamer-binding transcription factor 1.
Olf-1	× (0.0404)	×	Olfactory neuron-specific factor.
Sp1	× (0.0313)		Stimulating protein 1. Ubiquitous zinc finger transcription factor.
Spz1	× (<.0001)	×	Spermatogenic leucine zipper protein 1.
STAT	× (0.0661)		Signal transducer and activator of transcription. Transcription factors in cytokine-mediated biological responses.
STAT1	× (0.0021)		—
TATA (01)	× (0.0015)		Cellular and viral TATA box elements.
TATA (C)	× (0.0345)		—
TAXCREB	× (0.0389)		Tax/CREB complex.
Tst-1	× (0.0628)		Pou domain transcription factor.
VBP		×	Vitellogenin gene-binding protein.

This table provides the list of the most significant transcription factors identified by enrichment analysis and RLQ analysis. The *p*-values (*z*-score tests) of the enrichment analysis are given. Known biological functions are also provided.

The advantage of using RLQ analysis over EA is that the interpretation based on a single biplot is direct. The biplot representation shows associations between the time course effect of MF and the activation of transcription factors. Using EA, one has to identify the most enriched TFBS, then determine the genes that carry these TFBS (as well as the nature of the dysregulation), and finally interpret the role of transcription factors in the experiment. Discrepancies between the two approaches are partly due to the univariate nature of EA.When using RLQ analysis, functional annotation terms are treated in a multivariate fashion. This ensures that the presence of possible interactions and co-variations is accounted for in the analysis.

#### Other ordination methods

The following 3 ordination methods were compared to RLQ: 

● Correspondence analysis with projection of supplementary information on both rows and columns

● Between-group coinertia analysis (BG-COI)

● *L*-PLSR

**Figure 5:**
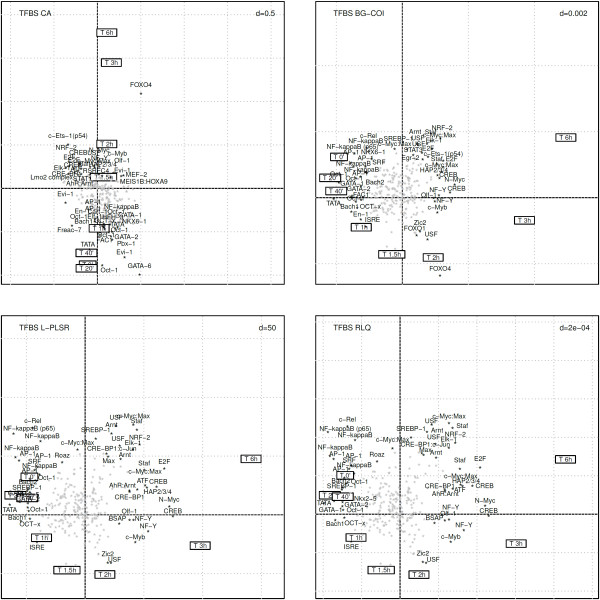
**Comparison of RLQ analysis with 3 alternative ordination methods to explore the transcription factor activity in the time course effect of mometasone furoate.** The upper left panel displays the biplot representation of correspondence analysis (CA) where supplementary information on both rows and columns were projected. The upper right panel displays the biplot representation of between-group coinertia analysis (BG-COI). The lower left panel shows the biplot representation of *L*-PLSR. The lower right panel depicts the RLQ analysis biplot.

Correspondence analysis (Figure 5, upper left panel), due to its unsupervised nature, does not allow to depict appropriately the time course effect of MF. The time gradient is only supported by the second axis and the expected early inhibition of key transcription factors such as NF- *κ*B combined with AP-1 and c-Rel is not immediately accessible in contrast to the results obtained by RLQ (Figure 5, lower right panel). When comparing the 50 most contributing TFBS identified by CA and RLQ, an overlap of 26 TFBS (52%) was found.

Between-group coinertia analysis (BG-COI) only differs from RLQ in the way tables **R** and **L** are linked together (in a symmetrical way for RLQ, and in an asymmetrical way for BG-COI). The results of BG-COI shown in Figure 5 (upper right panel) are mostly congruent with the ones obtained by RLQ. The biological findings derived by both methods are comparable. Among the 50 most contributing TFBSs identified by BG-COI and RLQ, an overlap of 40 TFBSs (80%) was found. Mathematically, RLQ provides a more general solution to the 3-table problem with a stronger theoretical background.

*L*-PLSR differs from RLQ in the sense that the central table is treated with a double centered PCA, whereas in RLQ the main table is treated by CA. In practice, due to the double-centering performed in *L*-PLSR, both methods provided almost identical results apart from a scaling factor (Figure 5, lower left panel). All of the 50 most contributing TFBS identified by *L*-PLSR match the ones identified by RLQ. However, to our knowledge, no implementation of *L*-PLSR adapted to genomics data is currently available.

Overall, the comparison of RLQ with other ordination methods stress the benefit of actively integrating external annotations on rows and columns using supervised modeling. Important data structures proved to be more challenging to interpret using unsupervised approaches. Both BG-COI and *L*-PLSR provide solutions which are in agreement with RLQ analysis. RLQ includes key features for life scientists, such as an available implementation of the method itself, together with a series of graphical tools, as well as various permutation procedures (Monte-Carlo permutation test, fourth-corner statistic), and several additional advanced procedures (*e.g.* between/within-class RLQ).

## Discussion

To date, several approaches have been proposed to interpret gene expression microarray data using external information. The classical approach identifies a list of genes of interest, then interprets these genes in a second step using tools of functional annotations. CA is a powerful method to describe sources of variation present in a microarray data set. Using a biplot representation, it is easy to simultaneously visualize the ordination of samples and to identify genes that are responsible for this ordination. It may be useful to include gene annotations directly in the frame of correspondence analysis. External information can be inserted by simple projection into the dataset in an unsupervised fashion as demonstrated by various authors [11,12]. Although this procedure brings insights which are helpful for the interpretation of the data, in most existing studies this additional information is only indirectly involved in the modeling of the data. In our example, we showed that this unsupervised approach was only partially effective, the interpretation of the data in the light of transcription factor activity remaining challenging.

Supervised counterparts of correspondence analysis were proposed in the literature [13,14]. Jeffery and collaborators [15] also described a method (BG-COI) integrating the information of TFBSs by combining in a 2-table scheme the gene expression data set (primarily analyzed by supervised non-symmetric correspondence analysis) with a TFBS occurrence table (primarily analyzed by principal component analysis). The main difference with the RLQ procedure is that in BG-COI, the first two matrices (**R** and **L**) are combined using a non-symmetrical procedure (between-class correspondence analysis), whereas the third table **Q** (TFBS occurrence) is integrated using a symmetrical procedure (co-inertia analysis). The initial asymmetric procedure necessitates a regression step implying dimensionality constraints in the number of experimental variables which can be integrated in the analysis [35]. In simple cases, *e.g.* when only one single categorical variable is used, this does not constitute a limitation. Accordingly, when applying BG-COI to the MF dataset, the results were comparable to the one obtained by RLQ with only minor discrepancies. However, in some other cases, for example when using a larger set of experimental variables, asymmetric procedures will show limitations.

RLQ generalizes the concept of symmetric analysis, an extension of the co-inertia principle for 3-tables, where each of the three tables plays independently a symmetric role. As highlighted by Dray and collaborators [19], the simultaneous analysis of this 3-table scheme is a more coherent solution. The advantage of RLQ over other methods such as between-class co-inertia analysis is that it provides a more consistent analysis framework with a stronger mathematical background. Other asymmetric approaches have been proposed in psychometrics such as the *double constrained correspondence analysis*[36] where the correspondence analysis of the central table **L** is constrained by both information on observations (table **R**) and variables (table **Q**). This double constrained procedure introduces 2 dimensionality constraints. Each regression step may lead to numerical instability that can generate poor predictive power. The solution proposed in RLQ analysis is more satisfying because it has no dimensionality restrictions, providing greater numerical stability particularly when the number of variables taken into account is large [18]. In RLQ, the central table is treated by correspondence analysis, whereas the 2 additional matrices can be analyzed using different schemes depending on the nature of the descriptive variables. The chemometrics *L*-PLSR procedure described by Martens and collaborators [20] follows the same idea with the only noticeable difference that the central table is treated by *principal component analysis* (after initial double-centering transformation), and not *correspondence analysis*. As previously shown, *L*-PLSR and RLQ are practically comparable and show very minor differences. Considering the different options associated with the analysis of the 2 marginal tables, and the choice in the scheme of the central table, this further extends the theoretical framework of RLQ.

In the scope of the current work, we described three applications of RLQ for the interpretation of high-throughput gene expression data, using TFBS, GO and KEGG pathways annotations as input of the **Q** matrix. Other variations around the same principle include the integration of literature co-occurrence information (*e.g.* by extracting information from *pubmed*), the presence/absence of microRNA targets [37], as well as more direct information such the chromosomal location, tissue expression patterns, *etc.*

It is worth noting that the transcription factors activity as assessed by RLQ analysis depends on several assumptions made for the identification of the putative TFBSs. This includes the choice of the TFBS motif database, the length of the promoter region, the threshold chosen to define the likelihood of the actual presence of TFBSs, *etc*. The current method takes into account the fact that several transcription factors can bind to a promoter and interact. However, more complex phenomena influencing the action of transcription factors, such as folding of the DNA promoter region [38], are not modeled in the current approach.

RLQ analysis is essentially exploratory. However, several testing procedures were specifically proposed in the framework of RLQ [19,23]. Using permutational models, the link between experimental variables and gene annotations can be tested. These inferential techniques provide an immediate overview of the nature (positive/negative) and significance of the relationship between experimental variables and gene annotations.

## Conclusion

RLQ analysis is a new approach to extract and visualize structures in a microarray dataset by combining external information on both columns (experimental variables) and rows (gene annotations). Biplot representations provide a unique all-integrated picture of the results, which allows us to directly relate experimental variables to gene annotations.

This approach was successfully used to describe the transcription factor activity associated with the action of the glucocorticoid MF furoate on the growth of human fibroblasts. In an integrated manner, RLQ analysis unveiled distinct mechanisms of action of glucocorticoids, in agreement with prior existing knowledge from the literature. The nuclear expression levels of OCT-1 and CREB confirmed the transcription factor activity predicted by using RQL analyses, and provide a direct molecular biological validation of the method.

The set of R functions proposed in the frame of this work further facilitates the use of RLQ analysis with regards to transcriptomics data in the light of GO, KEGG and TFBS information, as exemplified in this study.

Further work is needed to explore the performance of RLQ in specific cases, such as datasets comprising a larger set of experimental variables, or variables of heterogeneous nature (*e.g.* mixture of quantitative and qualitative variables). Computationally, the current implementation of RLQ allows to analyze efficiently standard gene expression datasets. However, further optimizations might be needed in order to deal with even larger highly multivariate datasets such as the ones generated by whole genome exon arrays.

## Competing interests

The authors declare that they have no competing interests.

## Authors’ contributions

FB wrote the manuscript, carried out the analysis and implemented the methodology. LK reviewed the manuscript and provided expertise on the clinical aspects. MB designed the experiments, and supervised all research experiments. JR performed the cell culture experiment and RNA extraction for gene expression analysis. PB, NM performed the Western analysis and validated the biological relevance of the findings. All authors read and approved the final manuscript.

## Supplementary Material

Additional file 1 Exploring the transcription factor activity in high-throughput gene expression data using RLQ analysis. Click here for file
