# Supplementary material for: Exploring the transcription factor activity in high-throughput gene expression data using RLQ analysis
Source: BMC Bioinformatics. 2013 Jun 6;14:178. doi: 10.1186/1471-2105-14-178 (PMC3686578; doi:10.1186/1471-2105-14-178)

Exploring the transcription factor activity in high-throughput gene expression data using RLQ analysis

Florent Baty, Jochen Rüdiger, Nicola Miglino,
Lukas Kern, Peter Borger, and Martin Brutsche

Appendix — Figures

Figure 1: **RLQ analysis of the time course effect of mometasone-furoate — fourth-corner statistics.** The significance of the link between experimental variables and transcription factor binding sites was tested by permutation procedures. All 282 TFBSs are represented row-wise in alphabetic order, whereas all time points (in hours) are represented column-wise. The following color coding was used: white for non-significant, light grey for negative significant, and dark grey for positive significant relationship.

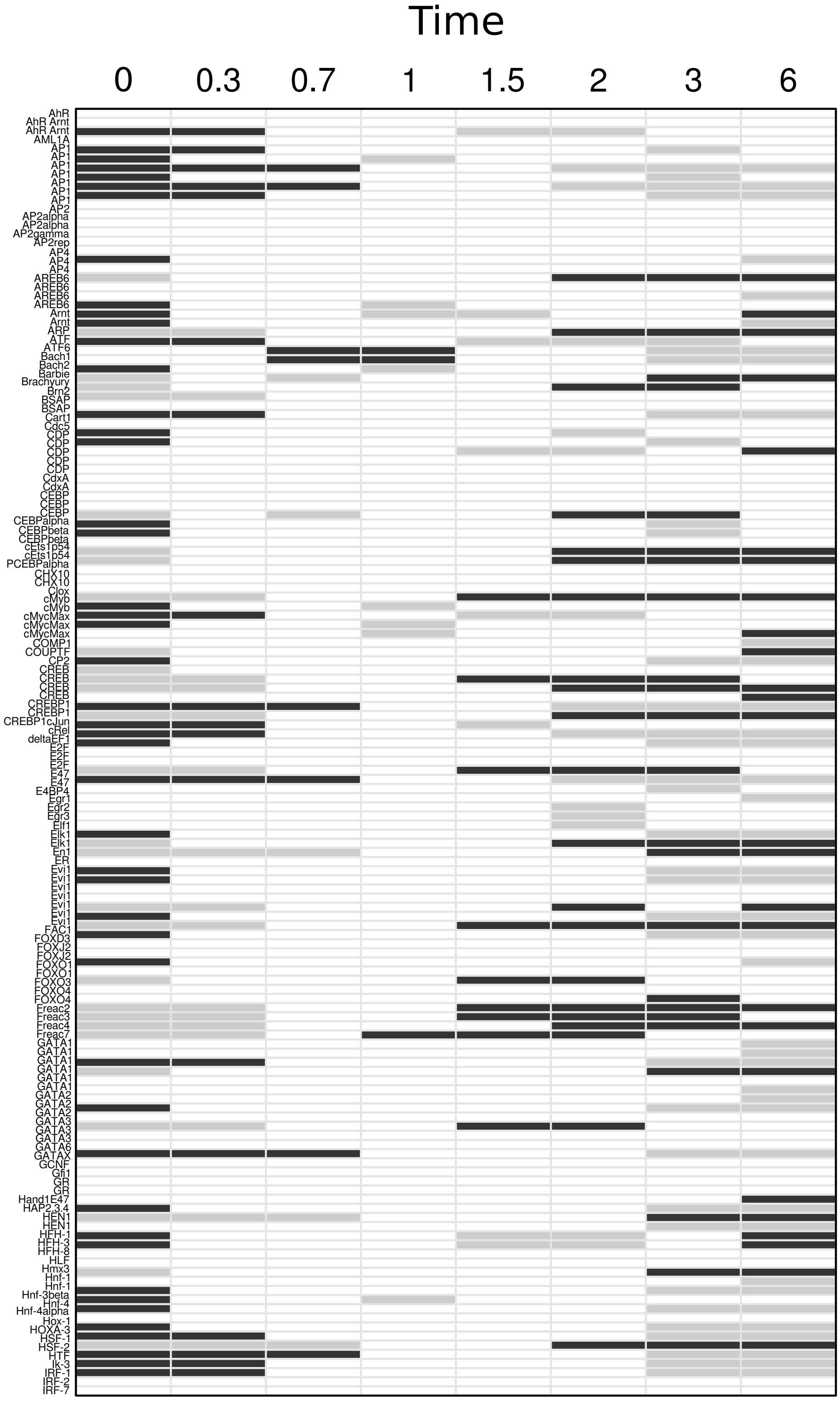

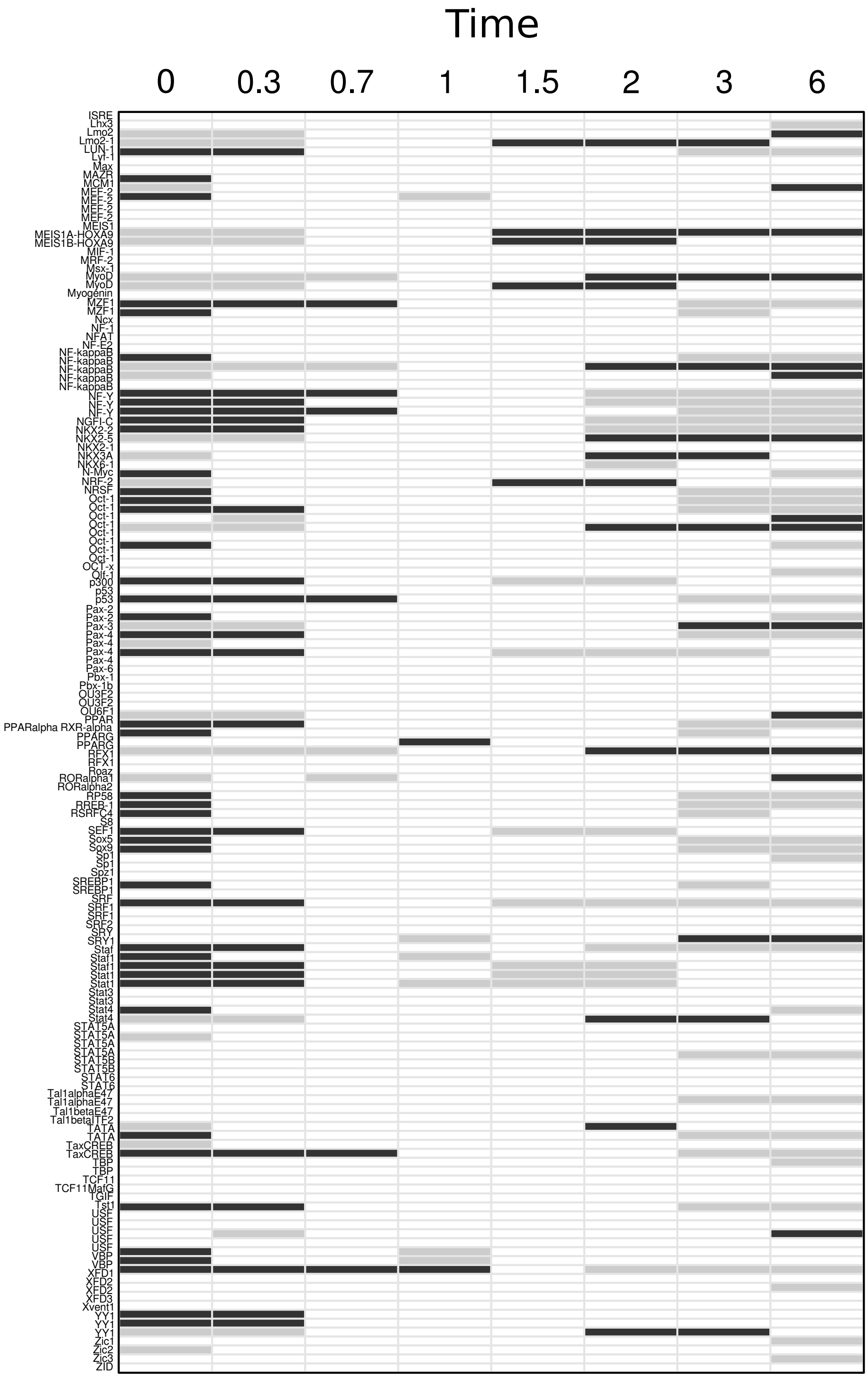
Time
0
0.3
0.7
1
1.5
2
3
6
ISRE
Lhx3
Lmo2
Lmo2-1
LUN-1
Lyl-1
Max
MAZR
MCM1
MEF-2
MEF-2
MEF-2
MEF-2
MEIS1
MEIS1A-HOXA9
MEIS1B-HOXA9
MIF-1
MRF-2
Msx-1
MyoD
MyoD
Myogenin
MZF1
MZF1
Ncx
NF-1
NFAT
NF-E2
NF-kappaB
NF-kappaB
NF-kappaB
NF-kappaB
NF-kappaB
NF-Y
NF-Y
NF-Y
NGFI-C
NKX2-2
NKX2-5
NKX2-1
NKX3A
NKX6-1
N-Myc
NRF-2
NRSF
Oct-1
Oct-1
Oct-1
Oct-1
Oct-1
Oct-1
Oct-1
Oct-1
OCT-x
Olf-1
p300
p53
p53
Pax-2
Pax-2
Pax-3
Pax-4
Pax-4
Pax-4
Pax-4
Pax-6
Pbx-1
Pbx-1b
OU3F2
OU3F2
OU6F1
PPAR
PPARalpha RXR-alpha
PPARG
PPARG
RFX1
RFX1
Roaz
RORalpha1
RORalpha2
RP58
RREB-1
RSRFC4
S8
SEF1
Sox5
Sox9
Sp1
Sp1
Spz1
SREBP1
SREBP1
SRF
SRF1
SRF1
SRF2
SRY
SRY1
Staf
Stat1
Stat1
Stat1
Stat1
Stat3
Stat3
Stat4
Stat4
STAT5A
STAT5A
STAT5A
STAT5A
STAT5B
STAT5B
STAT6
STAT6
Tal1alphaE47
Tal1alphaE47
Tal1betaE47
Tal1betaITF2
TATA
TATA
TaxCREB
TaxCREB
TBP
TBP
TCF11
TCF11MafG
TGIF
Tst1
USF
USF
USF
USF
USF
VBP
VBP
XFD1
XFD2
XFD2
XFD3
Xvent1
YY1
YY1
YY1
Zic1
Zic2
Zic3
ZID

Figure 2: **Western blot analysis of OCT-1 and CREB protein expression levels.** This shows the time course effect of MF on the expression levels of nuclear (left lanes) and cytosolic (right lanes) OCT-1 and CREB in primary lung fibroblasts.

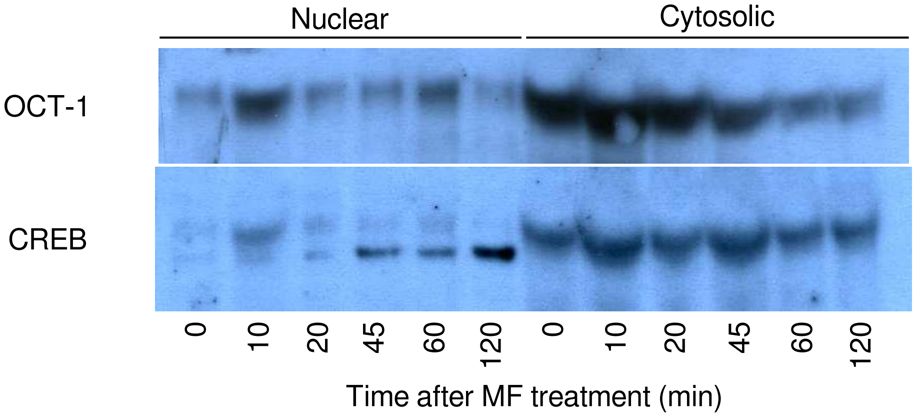

Supplement: Additional file 1 — Exploring the transcription factor activity in high-throughput gene expression data using RLQ analysis. [file 1471-2105-14-178-S1.pdf]
